# Male sex increases the risk of diabetic retinopathy in an urban safety-net hospital population without impacting the relationship between axial length and retinopathy

**DOI:** 10.1038/s41598-022-13593-4

**Published:** 2022-06-13

**Authors:** Jing Qian, Zeeshan Haq, Daphne Yang, Jay M. Stewart

**Affiliations:** 1 University of California, San Francisco, Department of Ophthalmology, 490 Illinois Street, Floor 5, San Francisco, CA 94143-4081 USA; 2 Zuckerberg San Francisco General Hospital and Trauma Center, Department of Ophthalmology, San Francisco, CA USA; 3 Children’s Hospital of Nanjing Medical University, Nanjing, China

**Keywords:** Diabetes complications, Retinal diseases, Refractive errors

## Abstract

This study sought to assess the association between axial length (AL) and diabetic retinopathy (DR) in a diverse cohort of patients and to investigate the impact of sex on this relationship. An urban safety net hospital database was used for this cross-sectional observational study. Diabetic patients who underwent fundus photography and AL measurement between March 2017 and June 2020 were included. The fundus photographs were graded following the Early Treatment of Diabetic Retinopathy Study criteria. The study enrolled 1843 patients with diabetes (mean age: 56.9 ± 12.1 years; AL: 23.56 ± 1.12 mm), including 931 men and 912 women. Male sex was a risk factor for diabetic retinopathy (*P* = 0.001; odds ratio [OR] 1.5, 95% confidence interval [CI] 1.18–1.98). A higher DR prevalence was associated with shorter AL both in men (*P* = 0.003; OR 0.77; 95% CI 0.66–0.91) and women (*P* = 0.02; OR 0.83; 95% CI 0.71–0.97) after adjusting for systemic risk factors using multivariable logistic regression. There was no significant impact of sex on the relationship between AL and DR (*P* = 0.56). In the subset of patients with asymmetric DR, the percentage of patients whose shorter eye had a higher stage of DR was not significantly different between men and women (*P* = 0.20). Male sex is a risk factor for DR in a diverse safety-net hospital population. Longer AL is associated with a lower risk of DR, and this relationship is not affected by sex.

## Introduction

Diabetic retinopathy (DR) is a common complication of diabetes and remains the leading cause of preventable blindness across the world^1^. In a meta-analysis of 35 population-based studies from Asia, Australia, Europe, and the U.S., Yau et al. estimated the prevalence of DR to be 34.6% among people with diabetes^2^. In addition, the global case burden of people with diabetes continues to increase and is projected to reach 366 million by 2030^3^.

Epidemiologic studies have identified numerous risk factors associated with the development of DR including duration of diabetes, hyperglycemia, hypertension, hyperlipidemia, and obesity^4–7^. Notably, biological sex has also been implicated; specifically, male sex has been found to be an independent risk factor for DR in Western populations^8–10^. The mechanisms that underlie this association are unclear; however, sex hormone-related pathways might play a role.

There is an increasing body of evidence that suggests that a longer axial length (AL) confers protection against the development of DR^11–16^. One proposed mechanism for this observation is retinal capillary bed protection from increased pressure attenuation in longer retinal arterioles found in myopic eyes^17^. Diabetes causes dysfunction in vascular autoregulation^18^, which may render capillaries more susceptible to damage from high upstream pressure. Biological sex may impact this mechanism through alterations in ocular blood flow regulation mediated by sex hormones^19^. This explanation may account for some of the observed sex-based differences in DR.

Herein we evaluated the impact of sex on the relationship between AL and the presence of DR in a large, diverse cohort of patients.

## Methods

Institutional review board approval was granted by the University of California, San Francisco (UCSF). Research adhered to the tenets of the Declaration of Helsinki. UCSF granted a waiver of informed consent due to the study’s minimal risk.

Patients with type 1 or type 2 diabetes evaluated at the Zuckerberg San Francisco General Hospital and Trauma Center between March 2017 and June 2020 were included in this cross-sectional study. Exclusion criteria included (1) significant cataract or other media opacities; (2) history of ocular trauma; (3) non-diabetic vitreomacular disease; (4) previous ocular surgery except uncomplicated cataract surgery. Relevant demographic and clinical information were collected, including age, sex, race, duration of diabetes, body mass index (BMI), most recent hemoglobin A1c (HbA1c), history of any tobacco use, and presence of comorbid systemic disease (e.g. hypertension and hyperlipidemia). Duration of diabetes was determined by patient self-report and/or medical record review as previously reported^20^. Race was categorized following the classification recommended by the National Institutes of Health^21^.

Only one eye from each patient was included in this study. The right eye was chosen unless the patient had asymmetric DR, in which case the eye with more severe DR was chosen.

Ultra-widefield fundus photography (Optos Daytona; Optos PLC, Dunfermline, United Kingdom) was obtained for all patients and was evaluated by the department DR reading center. DR was categorized as none, mild non-proliferative DR (NPDR), moderate NPDR, severe NPDR, and proliferative DR (PDR) by Early Treatment of Diabetic Retinopathy Study (ETDRS) criteria^22^.

AL was measured by noncontact partial coherence laser interferometry (IOLMaster version 3.01; Carl Zeiss Meditec AG, Jena, Germany).

Descriptive and inferential statistics were performed with Stata version 16.0 (StataCorp LP, College Station, TX, USA). Continuous outcomes are presented as means ± standard deviations and compared using Student’s t tests. Categorical variables are presented as percentages and compared using chi-square tests. The effect of sex on the relationship between AL and DR was evaluated using multivariable logistic regression. Previously established systemic risk factors for DR were included as covariates in the regression analysis^4–10^. A *P* value less than 0.05 was considered statistically significant; however, Bonferroni corrections were applied when appropriate.

### Ethics approval and consent to participate

Institutional review board approval was obtained from the UCSF Human Research Protection Program, #17-21703. A waiver of consent to participate was obtained since the study was a retrospective review.

## Results

After the application of exclusion criteria, 1843 patients with diabetes were studied and, of these, 931 (50.5%) were men (Table 1). An overwhelming majority (1813 patients) had type 2 diabetes. The women were older (*P* < 0.001) and had a different racial composition (*P* < 0.001). In addition, their HbA1c levels were lower (*P* < 0.001) and they were less likely to report a history of smoking (*P* < 0.001). The prevalence of DR was 28.9% and 24.2% amongst men and women, respectively, which was not significantly different (P = 0.024, not significant after Bonferroni correction). Supporting information is available online as supplemental material (S1 Table).

**Table 1:** Demographic and clinical characteristics of patients.

Characteristic	All patients (n = 1843)	Men (n = 931)	Women (n = 912)	*P* value
**Age (years)**	56.9 ± 12.1	55.6 ± 11.8	58.3 ± 12.3	< 0.001
**Race**				< 0.001
White or Caucasian	170 (9.2)	119 (12.8)	51 (5.6)	
Black or African American	153 (8.3)	95 (10.2)	58 (6.4)	
Hispanic	952 (51.7)	464 (49.8)	488 (53.5)	
Asian	496 (26.9)	215 (23.1)	281 (30.8)	
American Indian or Alaskan Native	9 (0.5)	4 (0.4)	5 (0.5)	
Native Hawaiian or Pacific Islander	19 (1.0)	7 (0.8)	12 (1.3)	
Other	44 (2.4)	27 (2.9)	17 (1.9)	
**Duration of diabetes (years)**	7.52 ± 7.36	7.10 ± 7.12	7.79 ± 1.95	0.015
**Body mass index (kg/m**^**2**^**)**	30.51 ± 6.94	30.23 ± 6.71	30.79 ± 7.15	0.081
**Hemoglobin A1c (%)**	8.01 ± 2.18	8.22 ± 2.36	7.79 ± 1.95	< 0.001
**Hypertension**	1150 (62.4)	568 (61.0)	582 (63.8)	0.214
**Hyperlipidemia**	1034 (56.1)	502 (53.9)	532 (58.3)	0.056
**Past smoker**	527 (28.6)	412 (44.3)	115 (12.6)	< 0.001
**Diabetic retinopathy**	490 (26.6)	269 (28.9)	221 (24.2)	0.024
Diabetic retinopathy grade*				0.045
None	1353 (73.4)	662 (71.1)	691 (75.8)	
Mild NPDR	352 (19.1)	185 (19.9)	167 (18.3)	
Moderate NPDR	97 (5.3)	60 (3.3)	37 (4.1)	
Severe NPDR	19 (0.7)	9 (0.5)	10 (1.1)	
PDR	22 (1.2)	15 (0.8)	7 (0.8)	
Axial length (mm)*	23.56 ± 1.12	23.91 ± 1.01	23.20 ± 1.12	< 0.001

Data are presented as a mean ± standard deviation or raw counts (percentage) as appropriate for each variable.

*NPDR* non-proliferative diabetic retinopathy, *PDR* proliferative diabetic retinopathy.

*The eye laterality selection process is described in the methods section.

Table 2 summarizes results from multivariable logistic regression modeling with the presence of DR as the outcome. An increased risk of DR was associated with male sex (*P* = 0.001), longer duration of diabetes (*P* < 0.001), higher HbA1c (*P* < 0.001), hypertension (*P* = 0.002), and shorter AL (*P* = 0.020 in women, and *P* = 0.003 in men). There was no significant interaction between sex and AL on the risk of DR (*P* = 0.555). A 1 mm increase in AL was associated with a 17–23% reduction in DR prevalence after adjusting for sex and other risk factors.

**Table 2:** Results of multivariable logistic regression with diabetic retinopathy as the dependent variable.

Independent variable	OR	95% CI	*P* value
**Age**	0.993	0.982–1.004	0.208
**Male sex**	1.531	1.182–1.983	0.001
**Duration of diabetes**	1.089	1.072–1.107	< 0.001
**Past smoker**	1.001	0.767–1.307	0.993
**Hemoglobin A1c**	1.207	1.146–1.270	< 0.001
**Body mass index**	0.982	0.964–1.000	0.045
**Hypertension**	1.534	1.172–2.007	0.002
**Hyperlipidemia**	1.049	0.824–1.335	0.698
**Race**			
White or Caucasian	Reference	Reference	Reference
Black or African American	0.626	0.359–1.090	0.098
Hispanic	0.929	0.619–1.393	0.722
Asian	0.894	0.578–1.385	0.617
American Indian or Alaskan Native	3.099	0.724–13.263	0.127
Native Hawaiian or Pacific Islander	1.035	0.338–3.170	0.952
Other	0.455	0.182–1.143	0.094
**Axial length (women)**	0.829	0.709–0.970	0.020
**Axial length (men)**	0.774	0.656–0.914	0.003

*OR* odds ratio, *CI* confidence interval.

Of patients with any stage of DR in one eye, 216 (men = 120, women = 96) had asymmetric DR where one eye had a different stage of DR than the other. In this group, most patients demonstrated a single grade difference between their eyes (i.e. no DR and mild NPDR, moderate NPDR and severe NPDR), and the distribution of these differences was not significantly different between men and women (P = 0.318). In addition, the inter-eye AL difference was similar for both sexes (P = 0.894). The proportion of patients whose shorter eye had worse DR was not significantly different between sexes (*P* = 0.202).

## Discussion

This cross-sectional study of 1843 diabetic patients seen at an urban safety net hospital investigated risk factors for the development of DR. Male sex, duration of diabetes, HbA1c, hypertension, and AL were associated with DR, while race demonstrated no significant impact. These findings are consistent with prior Western population-based and multi-ethnic studies^8,9,22^. In addition, a 1 mm increase in AL was associated with a 17–23% reduction in DR prevalence after adjusting for sex and other risk factors. This inverse association between AL and DR has significant public health implications given the ongoing global myopia pandemic. Interestingly, this finding is almost identical to the results reported from a similar cohort study based in northern China^16^. Most studies concerning myopia and DR have included populations that were restricted to a single racial or ethnic group^11,13,16,23^. Our study improved upon this work through the inclusion of a larger and more diverse patient population that consisted of several different racial groups. To our knowledge, this study involves the largest multi-racial diabetic cohort with AL measurements to date.

Many studies have reported a protective effect of myopia^11,15,24,25^ on DR. In particular, globe elongation is felt to mediate this association^11,13–15^. Multiple mechanisms for this association have been proposed. Longer retinal arterioles in these larger-than-normal eyes can lead to increased pressure attenuation, which may confer some degree of protection on the retinal capillary bed^17^. Jonas et al. revealed that the intraocular concentration of vascular endothelial growth factor (VEGF) decreased significantly with increasing AL^26^. In addition, the vitreous is less viscous in axially elongated eyes, which may be associated with a faster turnover of VEGF out of the eye^16^. Moreover, posterior vitreous detachments are more prevalent in patients with longer ALs^24^, and are protective against the formation of neovascularization in proliferative DR^27^.

We found that male sex was a risk factor for DR in this large, multi-ethnic study population. This is consistent with numerous prior studies that have also identified this association^8–10^. The mechanisms of sex differences in diabetic microvascular complications are unknown. Biological differences, such as sex hormones, may modulate the retinal damage in diabetes^28^. For example, it is known that changes in circulating sex hormone levels are thought to play a role in the progression of DR during pregnancy^29^. Inflammatory cytokine profiles may differ according to sex, which could also influence DR development and progression^30^. In addition, sex differences in behavior and treatment between men and women may play a role; lifestyle choices such as smoking that can influence vasculopathy may be more prevalent in men. Treatment adherence may also differ between men and women.

In this study, we utilized the availability of AL measurements in our patients to assess the impact of sex on the relationship between AL and DR, since sex and AL each independently impact the risk of DR. In this series, sex did not impact the relationship of AL with DR, as assessed by interaction analysis with logistic regression modeling. In addition, in the subset of patients with asymmetric DR, no sex-based difference was seen in the frequency of worse DR grade occurring in the eye with a shorter AL. This latter analysis in patients with asymmetric DR theoretically controls for all known and unknown confounders since both eyes reside within the same patient. Taken together, all of these findings suggest that other factors, beyond AL, must contribute to the observed sex-based differences in DR.

This study has several limitations. First, men and women differed significantly with regards to a number of characteristics (see Table 1); however, many of these differences were not clinically significant. In addition, information on menopausal status and use of hormone replacement therapy was not solicited. The use of a cross-sectional design precludes causal inference. Lastly, residual confounding may have been present in the multivariable logistic regression.

In conclusion, our study confirmed that male sex was a risk factor for DR, but we found no association between sex and the effect of AL on DR development. Future studies are needed to evaluate the mechanisms underlying sex-based differences in the risk of DR and the inverse relationship between DR and AL.

## Perspectives and significance

This study capitalized on the availability of a large dataset at an urban, safety-net hospital in which patients with diabetes underwent collection of both fundus photographs and AL measurements. Our group wished to investigate sex differences in DR by assessing the impact of sex on the relationship between AL and DR risk. While the findings confirmed the hypothesis that male sex is a risk factor for DR, we found that the protective effect of AL on DR did not manifest differently according to sex. The lack of interaction between sex and AL suggests that they could affect DR through two entirely different mechanisms, a possibility that bears further investigation.

## Supplementary Information

Supplementary Information.

## Data Availability

The dataset generated during the current study is included with this report as supplemental material.
